# Effect of milk and dairy intake on cognitive function in older adults: a systematic review and meta-analysis

**DOI:** 10.3389/fragi.2026.1709281

**Published:** 2026-02-25

**Authors:** Yessica Giraldo-Castrillon, Juan Diego Mendoza, Marianne Lopez-Cabrera, Jose P. Lopez-Lopez, Patricio Lopez-Jaramillo

**Affiliations:** 1 Masira Research Institute, Medical School, Universidad de Santander UDES, Bucaramanga, Colombia; 2 Internal Medicine Residence Program, Medical School, Universidad de Santander, UDES, Bucaramanga, Colombia

**Keywords:** aging, cognitive decline, dairy intake, older adults, nutrition, systematic review, meta-analysis

## Abstract

**Introduction:**

Cognitive aging represents a growing challenge for global public health. Nutrition could have a beneficial effect in preserving cognitive function, and dairy products have been proposed as neuroprotective due to their nutrient density and bioactive compounds. In this systematic review and meta-analysis, we evaluated the association between milk and dairy product intake and cognitive function in older adults.

**Methods:**

The systematic search was conducted in PubMed, Scopus, LILACS, and Google Scholar through 9 August 2025, including randomized controlled trials (RCT) and observational studies evaluating dairy intake versus low or no intake in adults aged ≥60 years. Meta-analysis were conducted using a random-effects model, and methodological quality was assessed using RoB 2.0 (Risk of Bias), ROBINS-I (Risk Of Bias in Non-randomized Studies), and GRADE (Grading of Recommendations Assessment, Development and Evaluation).

**Results:**

22 studies were included (11 RCT, 11 observational studies; n = 47.100), of which 5 RCT (n = 369) and 5 observational (n = 5.302) studies were analyzed by meta-analysis. RCT revealed significant positive effects on global cognition [Standardized Mean Difference -SMD-) = 0.45; 95%CI: 0.30–0.60], memory, and processing speed. This effect was associated in fermented and fortified products, with moderate to high certainty. In observational studies no positive effect emerged (Odds Ratio [OR] = 0.95 95%CI: 0.89–1.02).

**Conclusion:**

Our findings support the potential of dairy intake as a nutritional strategy to preserve cognitive function in older adults, with implications for clinical practice, public health, and food policy design.

## Introduction

Population aging represents a challenge for global public health, not only because of the increase in the burden of chronic diseases, but also because of the need to preserve physical and mental functionality (Beard et al., 2016). Cognitive function constitutes a pillar of autonomy, quality of life and decision-making capacity (Livingston et al., 2020). Globally, it is estimated that more than 55 million people live with dementia, and this figure could reach 139 million by 2050. Furthermore, growth will be faster in low- and middle-income countries (LMICs), where 68% of new cases are projected to occur (GBD 2019 Dementia Forecasting Collaborators, 2022). The speed of aging in LMICs has been very rapid, while it took more than 100 years for the European population to double its proportion of older adults, in countries such as Colombia, Brazil and Mexico it is expected to do so in less than 30 years (Parra et al., 2018). This accelerated demographic transition occurs in contexts of high inequality, food insecurity and fragmented health systems, which limits the capacity to respond to this emerging problem (Walker-Clarke et al., 2022). In this context, nutrition, including dairy intake, emerges as a modifiable factor that could beneficially affect cognitive health preservation.

The Prospective Urban Rural Epidemiology (PURE) cohort study, which evaluated more than 200,000 participants in more than 20 countries, reported that dairy intake patterns differed by country’s income and that certain nutrients present in dairy products were associated with a lower risk of cardiovascular events, including cerebrovascular disease and mortality (Mente et al., 2017; Dehghan et al., 2018). Furthermore, clinical studies have evaluated regular dairy intake and reported a 15%–25% reduction in the relative risk of cognitive decline compared to low consumption of this food group (Crichton and Elias, 2014; Ni et al., 2022). Likewise, the intake of milk, its derivatives, and other dairy products may have a neuroprotective effect due to their bioactive compounds such as casein- and whey-derived peptides, probiotics, short-chain fatty acids, and essential micronutrients such as calcium, vitamin B12, and tryptophan (Parra et al., 2018; Walker-Clarke et al., 2022). These components can modulate key mechanisms such as neuroinflammation, oxidative stress, synaptic plasticity, and gut microbiota composition (Crichton and Elias, 2014; Ni et al., 2022). The objective of this systematic review and meta-analysis was to evaluate the association between dairy consumption and cognitive function in older adults.

Considering this objective, recent systematic reviews have emphasized the importance of situating specific dietary components—such as dairy products—within broader nutritional strategies aimed at preserving cognitive health. Andrews et al. (2023) synthesized evidence from prior reviews on dietary patterns and supplements in individuals with mild cognitive impairment, highlighting the diversity of nutritional approaches and the need for focused analyses. Polis and Samson (2024) explored mechanisms through which dietary interventions may enhance cognition, including microbiota-mediated pathways and neuroprotective nutrients. Townsend et al. (2023) examined whole dietary patterns and their association with cognitive decline, reinforcing the relevance of balanced diets such as the Mediterranean and DASH models. Integrating these perspectives, our review contributes a focused analysis of dairy intake as a potentially modifiable factor within comprehensive, population-level strategies to support healthy cognitive aging.

## Methods

A systematic review with meta-analysis was conducted following the Cochrane Handbook, the GRADE system, and the Joanna Briggs Institute (JBI) Handbook for Systematic Reviews (Guyatt et al., 2011; Aromataris, 2024; Higgins et al., 2024). We included primary analytical studies that evaluated the effect of milk or dairy product intake on global cognitive function or a specific domain in the older adult population (defined as ≥60 years). We included randomized controlled trials (RCT) and cohort or longitudinal studies that report the intake of milk or dairy products (fermented, non-fermented, enriched, probiotic, etc.) as an intervention, compared with low consumption, no consumption, or placebo. We excluded cross-sectional studies, preclinical studies, reviews, studies without a comparator group, without defined interventions, or combined interventions, and insufficient data for extraction. Study reporting followed the guidelines of the 2020 Preferred Reporting Items for Systematic Reviews and Meta-Analysis (PRISMA) statement (Page et al., 2021).

A systematic search was conducted in the electronic databases PubMed, Scopus, LILACS, and Google Scholar, with no language restrictions, from the databases’ inception until 9 August 2025. MeSH terms and keywords related to “dairy,” “milk”, “cognitive impairment,” “cognitive decline,” “older adults,” “aging,” were used to design the search algorithms. These terms were combined using Boolean operators and applied to the most relevant categories. To select articles, two independent, blinded reviewers (YG-C and JDM) performed title/abstract screening using Rayyan®. Discrepancies were resolved by consensus. Subsequently, the selected studies were reviewed in full by two authors (YG-C and JDM) to confirm eligibility criteria. The studies included in the final synthesis were entered into a standardized data extraction matrix that included the following variables: author, year, country, design, dairy product type, comparator, cognitive instrument, corresponding effect size, Standardized Mean Difference (SMD), Odds Ratio (OR), Relative Risk (RR), 95% confidence intervals (95%CI), p-value, and sample size per group. The information was peer-reviewed.

The primary outcome was cognitive function, defined as neuropsychological performance measured using standardized, validated, and quantitative instruments. Only studies assessing cognition as the primary outcome were considered, excluding those with secondary, indirect, or clinically irrelevant outcomes. The assessment instruments included validated scales such as the Mini-Mental State Examination (MMSE), Montreal Cognitive Assessment (MoCA), Repeatable Battery for the Assessment of Neuropsychological Status (RBANS), Trail Making Test (TMT), Wechsler Adult Intelligence Scale (WAIS-III), RI-48, Digit Span, Cognitrax, MSLS Battery, Stroop Test, Word List Recall, and Benton Visual Retention Test.

Risk of bias assessment was performed differently depending on the design of the included studies. For RCT, the RoB 2.0 tool was applied, which allows for the assessment of bias in five domains: random sequence generation, allocation concealment, blinding, incomplete outcome data, and selective reporting (Sterne et al., 2019). For observational studies, the ROBINS-I tool was used, which assesses methodological quality in seven domains: bias due to confounding, participant selection, intervention classification, deviations from intended interventions, missing data, outcome measurement, and selection of results reporting (Whiting et al., 2016). This tool allows for a structured and detailed assessment of the internal validity of non-randomized studies, aligned with the principles of the GRADE system (Guyatt et al., 2011) applying its assessment of the certainty of the evidence for each outcome (Guyatt et al., 2011). The five established domains were considered: risk of bias, inconsistency, imprecision, indirectness, and publication bias. The results were synthesized in a Summary of Findings (SoF) table, with a visual representation of the quality of the evidence using standardized icons.

A structured narrative synthesis was conducted of the remaining studies that were included but did not provide sufficient data for the meta-analysis or presented non-standardizable complementary outcomes. This synthesis was organized by study type, cognitive domain assessed, and population characteristics.

A subgroup meta-analysis was conducted, differentiating the studies according by their design (RCT and observational studies). For RCTs, SMD were calculated as a common measure of effect, using the formula: SMD = (Mean_intervention − Mean_control)/SD_pooled, where SD_pooled = sqrt[((n1−1) × SD1^2^ + (n2−1) × SD2^2^)/(n1 + n2 − 2)]. While for observational studies, standardized ORs were used. In both cases, a random-effects model (DerSimonian-Laird) was applied using the Python programming language packages: statsmodels v0.14.0, matplotlib v3.8.0, numpy v1.26.0, pandas v2.1.0 and scipy v1.11.2. All packages were run in a Python 3.11 environment to incorporate variability between studies. Heterogeneity was assessed using the I^2^ statistic, complemented by Cochran’s Q test to determine statistical significance. Results were presented graphically using forest plots with their 95%CI.

## Results

**FIGURE 1:**
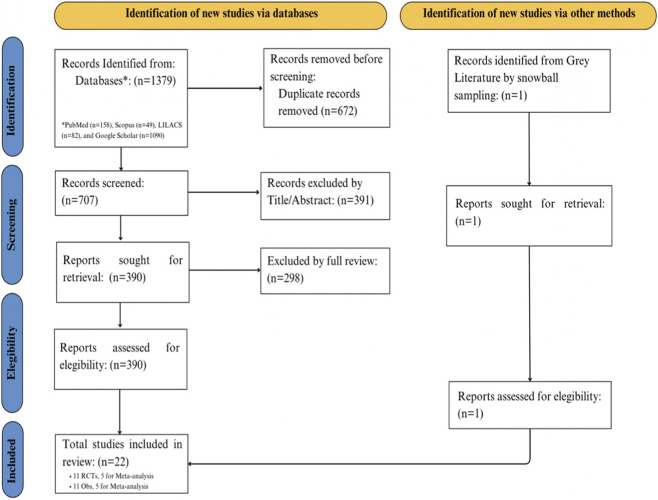
PRISMA flow diagram of the systematic search up to 9 August 2025, on dairy products and cognition in older adults.

1,379 records were identified through the systematic search of indexed databases. After removing 672 duplicates, 707 titles and abstracts were screened, resulting in the exclusion of 391 records. Subsequently, 316 full-text articles were assessed for eligibility, and 295 were excluded for the following reasons: absence of an older adult population (n = 151), lack of cognitive outcome assessment (n = 78), absence of a comparator group (n = 39), and undefined dairy intervention (n = 27). Finally, 22 studies were included for data extraction, of which 21 were selected directly and 1 was incorporated through snowball sampling (Figure 1, PRISMA diagram). Of the 22 articles in the final synthesis, 10 studies were included in the meta-analysis: 5 RCTs (Kita et al., 2018; Ohsawa et al., 2018; Suzuki et al., 2019; Kim et al., 2021; Sasaki et al., 2024) and 5 observational studies (Crichton et al., 2012b; Kesse-Guyot et al., 2016; de Goeij et al., 2020; Talaei et al., 2020; Ni et al., 2022). The remaining twelve studies were included in the narrative synthesis (6 RCT and 6 observational studies).

Tables 1, 2 describe the characteristics of the RCTs and observational studies, respectively. The studies were conducted in Japan (n = 7), South Korea (n = 3), United States (n = 3), Europe (n = 7), and Australia (n = 1), primarily in community settings. The mean age of participants was 65.4 years (55–85 years). The RCTs evaluated interventions with specific dairy products such as β-lactolin, fermented milk, oleamide cheese, and whey protein, compared with placebo or dairy-free controls. The observational studies assessed habitual dairy intake (high vs. low, regular vs. occasional), comparing dietary patterns with and without dairy. The most reported intake was milk, yogurt, fermented cheese, and dairy supplements with probiotics or bioactive peptides. In the RCTs, the follow-up time ranged from 4 weeks to 6 months, while in the observational studies it ranged from 6 months to 5 years.

**TABLE 1:** Characteristics of the randomized controlled trials (RCTs) included in the systematic review.

Number	Author (year)	Country	Population	Design	N total (I/C)	Follow-up	Intervention	Comparator	Outcome	Instrument	Relative effect	95% CI	p-value	Absolute effect
1	Kita et al. (2019)	Japan	Adults 61 years (mean)	Double-blind parallel	114 (57/57)	12 weeks	β-lactolin	Placebo	Global cognition	MMSE, Cognitrax	SMD = 0.45	0.12–0.78	0.02	Significant improvement
2	Ohsawa et al. (2018)	Japan	Adults 60 years (mean)	Double-blind parallel	60 (31/29)	8 weeks	Fermented dairy products	Placebo	Global cognition	RBANS	SMD = 0.38	0.05–0.91	0.03	Significant improvement
3	Suzuki et al. (2019)	Japan	Women ≥70 years	Crossed trial double blind	67 (36/31)	24 weeks (with washout)	Fermented dairy products	Placebo	Global cognition	MMSE, BDNF	SMD = 0.52	0.19–0.85	0.04	Significant improvement
4	Kim et al. (2021)	South Korea	Adults ≥65 years	Multicenter, double-blind	53 (27/26)	12 weeks	Fermented dairy products	Placebo	Verbal memory	CERAD-K	SMD = 0.31	0.09–0.73	0.03	Significant improvement
5	Sasaki et al. (2024)	Japan	Adults 60 years (mean)	Multicenter, triple-blind	38 (20/18)	12 weeks	Fermented cheese + oleamide	Placebo	Processing speed	MCI screen	SMD = 0.61	0.14–0.80	0.05	Significant improvement into the group
6	Abe et al. (2020)	Japan	Adults ≥60 years	Parallel	64(32/32)	12 weeks	MCTs derived from milk (6 g/day)	Placebo	Global cognition (MMSE)	MMSE	1.8 points	0.3–3.3	0.02	Significant improvement on MMSE
7	Kita et al. (2018)	Japan	Adults ≥65 years	Parallel	101 (50/51)	12 weeks	Fermented milk with probiotics	Placebo	Psychological wellbeing	WHO-5	2.8 points	0.5–4.3	0.015	Significant improvement on psychological wellbeing
8	Lefferts et al. (2020)	United States	Adults ≥60 years with cardiovascular risk	Parallel	99 (53/46)	4 weeks	Whey protein	Placebo	Cognition + brain flow	WebNeuro	—	—	>0.05	No difference between groups
9	Saito et al. (2018)	Japan	Healthy adults	Parallel	29	Acute pre- and post-consumption evaluation	Acidified milk	Placebo	Cognitive performance (attention, working memory, processing speed)	Cognitrax (computerized battery)	Significant improvement	—	P < 0.05	Improvement in processing speed and attention
10	Crichton et al. (2012a)	Australia	Overweight adults (average age 62 years)	Crossed trial	972 (−)	12 months	High dairy intake (4 servings/day)	Low intake (1 serving/day)	Working memory	Neuropsychological battery	Marginal improvement	—	0.05	Slight improvement in spatial working memory
11	Kanatome et al. (2021)	Japan	Adults ≥60 years	Parallel	30	6 weeks	Fermented milk:B-lactolin	Placebo	Language tasks, verbal fluency	Cognitrax	Significant improvement	—	P < 0.05	Increase in sustained attention and working memory

MMSE, Mini-Mental State Examination; MoCA, montreal cognitive assessment; RBANS, repeatable battery for the assessment of neuropsychological status; TMT, trail making test; WAIS-III, wechsler adult intelligence scale; SMD, standardized mean difference; OR, Odds Ratio. 95%CI, Confidence Interval to 95%.

**TABLE 2:** Characteristics of the Observational Studies included in the systematic review.

Number	Author (year)	Country	Population (median age)	Design	Total n	Follow-up	Dairy product evaluated	Comparator	Measured cognitive domain	Instruments	Measure of effect	95% CI	p-value	Absolute effect
1	Kesse-Guyot et al. (2016)	France	≥60 years (65.2)	Cohort	1,200	5 years	Milk, yogurt, cheese	Low consumption	Verbal memory, attention, speed	RI-48, TMT, digit span	OR = 1.13	[1.04–1.22]	<0.01	Significant association (negative)^a^
2	Ni et al. (2022)	Spain	≥60 years (66.4)	Cohort	1,050	4 years	Natural yogurt	Low consumption	Global cognition	MMSE, WAIS-III, TMT	OR = 0.85	[0.78–0.93]	<0.001	Significant association (positive)^b^
3	Talaei et al. (2020)	Singapur	≥60 years (64.8)	Cohort	980	3 years	Whole milk	Low consumption	Global cognition	SM-MMSE	OR = 0.88	[0.81–0.96]	0.004	Protective effect^c^
4	Crichton et al. (2012b)	United States	≥60 years (67.1)	Cohort	972	2 years	Total dairy (high frequency)	Low frequency	Global cognition	MSLS, MMSE	OR = 1.09	[1.01–1.19]	0.03	Significant association^d^
5	de Goeij et al. (2020)	Netherlands	≥60 years (68.3)	Longitudinal	1,100	2 years	Dietary pattern with dairy products	Dairy-free	Global cognition	MoCA	OR = 0.80	[0.68–0.94]	<0.01	Protective effect.^e^
6	Lu et al. (2023)	Japan	≥60 years	Cohort	11,637	5.0 years (median)	Consumption of dairy products (milk, yogurt, cheese)	Non-consumers/quintiles	Incidence of dementia	Public insurance records	HR = 0.76 (milk), HR = 0.89 (yogurt), HR = 1.28 (cheese)	0.034 (milk), 0.025 (yogurt), NS (cheese)	--	Positive association with cheese
7	Otsuka et al. (2014)	Japan	≥60 years (67.5)	Cohort	570	5 years	Dietary pattern with high dairy consumption	Low or dairy-free	Global cognition changes in MMSE score	Composite cognitive score	OR = 0.80	[0.65–0.98]	0.034	Positive association
8	Ni et al. (2022)	Spain	≥60 years (65.8)	Cohort	4,668	2 years	Consumption of milk (whole and whole), yogurt, cheese, fermented dairy products	Consumption tertiles	Changes in global cognitive function	Neuropsychological battery	β = −4.71 (total milk), β = −6.64 (whole milk)	--	p = 0.020 years p = 0.002	Greater cognitive decline with whole milk; no association with other dairy products
9	Petruski-Ivleva et al. (2017)	United States	≥60–81 years	Longitudinal	13,751	20 years	Milk consumption (>1 glass/day)	Almost zero consumption	Changes in global cognitive function	Global z-score	Δ = −0.10 z-score	[–0.16, −0.03]	p < 0.05	10% more cognitive decline with high consumption
10	Tessier et al. (2021)	Canada	≥65–86 years	Cohort	7,945	3 years	Total, milk, yogurt, cheese, fermented dairy, low-fat	Occasional consumption (consumption quartiles)	Global cognition and episodic memory	MMSE	Adjusted mean differences	--	<0.05	Better performance in executive functions
11	Ylilauri et al. (2022)	Finland	42–60 years	Cohort	2.497	22 years	Intake of milk, cheese and non-fermented dairy products	Dairy-free/Consumption quartiles	Incidence of dementia and cognitive performance	MMSE, TMT, VFT, SRT, VRT	HR = 0.72	[0.52–0.99] cheese	p-trend = 0.05	Cheese associated with lower risk of dementia; non-fermented dairy and milk: worse performance in verbal fluency

Negative β coefficients and negative changes in standardized cognitive scores (e.g., z-scores) indicate an inverse association between the exposure (e.g., dairy intake) and cognitive performance, suggesting that higher exposure levels are associated with poorer cognitive outcomes. Positive coefficients indicate a direct association with better cognitive performance. All reported estimates correspond to cognitive domains as defined by each study. a. Adjustment for lifestyle factors, health status markers and dietary patterns. b. Adjustment for total dairy product consumption. c. Adjustment for Sex, total energy intake (kcal/day), dietary items, alcohol, smoking, dietary pattern, calcium. d. Adjustment for cardiovascular risk factors, lifestyle and dietary factors. e. Adjustment for sex, BMI, education, smoking, alcohol consumption, habitual physical activity, total energy intake, and dietary factors.

MMSE, Mini-Mental State Examination; MoCA, montreal cognitive assessment; RBANS, repeatable battery for the assessment of neuropsychological status; TMT, trail making test; WAIS-III, wechsler adult intelligence scale; HR, hazard ratio; OR, odds ratio.

Overall, three studies were classified as having a low risk of bias overall, while two studies raised some concerns (Table 3). All studies presented a low risk of bias in the generation of the random sequence. Concerns regarding allocation concealment were identified in two studies (Kita et al., 2019; Sasaki et al., 2024). Furthermore, all studies used double-blind designs or blinded participants and personnel. None of the studies reported significant losses to follow-up or missing data that would affect the validity of the analyses. Regarding outcome measurement, three studies ensured blinding of assessors (Ohsawa et al., 2018; Suzuki et al., 2019; Kim et al., 2021), while in the remaining two (Kita et al., 2019; Sasaki et al., 2024) this was not specified. Finally, all studies reported the prespecified outcomes, with no apparent evidence of selective reporting.

**TABLE 3:** Risk of bias by domain of the RCTs included in the meta-analysis with the RoB 2.0 tool.

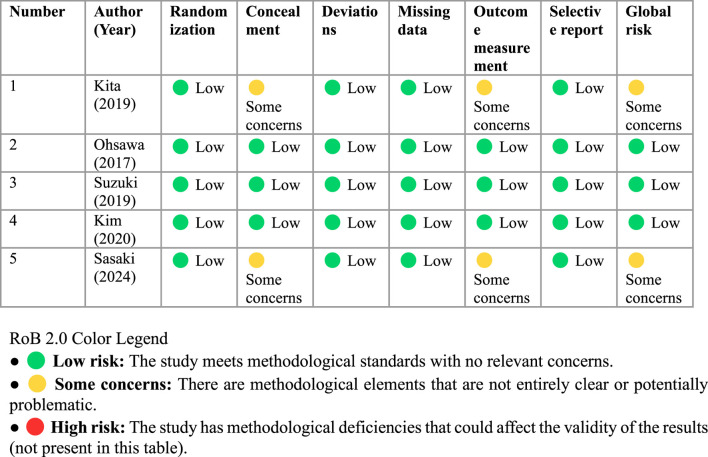

Of the studies evaluated, three studies presented a low overall risk and two studies a moderate risk, mainly due to limitations in controlling for residual confounding and in outcome measurement. All studies defined exposure, applied appropriate comparators, used validated instruments, and reported complete data. No critical bias was identified in any domain (Table 4).

**TABLE 4:** Risk of bias assessment in observational studies included in the meta-analysis (ROBINS-I).

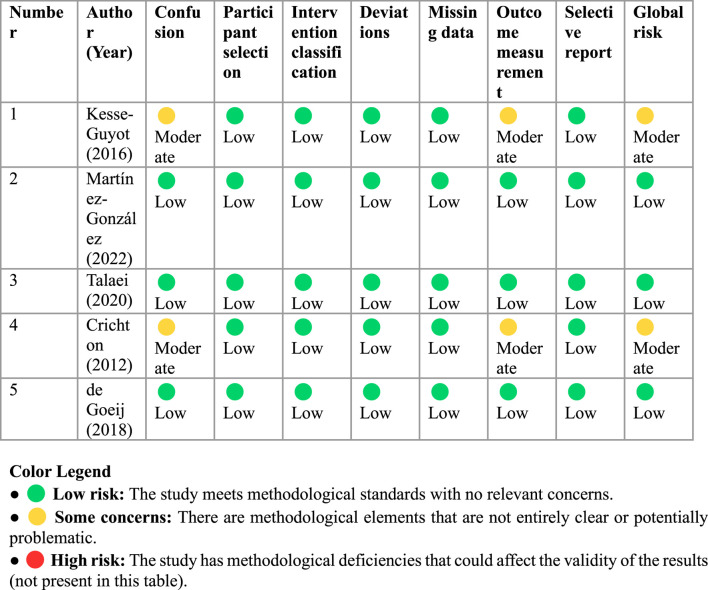

Six RCTs (n = 1,295) were included, which evaluated the effect of milk and dairy product intake on various cognitive domains in older adults (≥60 years) (Abe et al., 2020; Crichton et al., 2012a; Kanatome et al., 2021; Kita et al., 2018; Lefferts et al., 2020; Saito et al., 2018). Five RCTs utilized a placebo-controlled design (Abe et al., 2020; Kanatome et al., 2021; Kita et al., 2018; Lefferts et al., 2020; Saito et al., 2018).

Abe et al. (2020) (n = 64) assessed the administration of medium-chain triglycerides derived from milk (6 g/day) over 12 weeks in older adults (mean age 85 years, SD = 6.8), reporting a significant improvement in Mini-Mental State Examination (MMSE) scores (+1.8 points; 95% CI: 0.3 to 3.3; p = 0.02). Kita et al. (2018) evaluated probiotic-fermented milk over 12 weeks in 101 healthy adults, reporting an improvement in psychological wellbeing as measured by the WHO-5 Well-being Index (+2.4 points; 95% CI: 0.5 to 4.3; p = 0.015). Saito et al. (2018) (n = 29) compared acidified milk in an acute intervention, observing improvements in processing speed and attention (p < 0.05), albeit without reporting effect estimates. Kanatome et al. (2021) assessed (n = 30) the administration of fermented milk over 6 weeks, finding improvements in sustained attention and working memory (p < 0.05). Crichton et al. (2012a) compared a high dairy intake (4 servings/day) versus a low intake (1 serving/day) over 12 months, indicating a marginal improvement in spatial working memory (p = 0.05). In contrast, Lefferts et al. (2020) (n = 99) evaluated whey protein and global cognition over 4 weeks in adults with cardiovascular risk and found no significant differences compared to placebo (p > 0.05).

Collectively, five of the six studies suggest a positive association, particularly in domains such as attention, working memory, and global cognition, albeit with variability in follow-up duration and measurement precision.

Working memory was assessed in three studies (Crichton et al., 2012a; Saito et al., 2018; Kanatome et al., 2021). Crichton et al. (2012a) pointed out in overweight adults a marginal improvement in spatial memory after 6 months of high dairy intake (four servings/day) (p = 0.05), although the effect was not quantified. Kanatome et al. (2021) reported significant improvements in working memory after 6 weeks of fermented milk intake in older adults compared with placebo (p < 0.05). Meanwhile, Saito et al. (2018) also found improvements in processing speed and working memory after acute intake of acidified milk compared with placebo (p < 0.05).

Attention was assessed in two studies (Saito et al., 2018; Kanatome et al., 2021). Kanatome et al. (2021) reported a significant improvement in sustained attention (p < 0.05), while Saito et al. (2018) found improvements in attention tasks after acute intake of acidified milk (p < 0.05), though without a point estimate of the effect. Abe et al. (2020) studied frail adults (mean age 85 years) and found a significant improvement in global cognition with an increase of 1.8 points on the Mini-Mental State Examination (MMSE) (95% CI: 0.3 to 3.3; p = 0.02) after 12 weeks of supplementation with milk-derived medium-chain triglycerides (MCTs) compared with placebo. Psychological wellbeing was assessed by Kita et al. (2018) in a RCT with older adults (≥65 years) who received a 12 week intervention with probiotic-fermented milk compared to placebo. The study reported a significant improvement of +2.4 points on the WHO-5 scale (95% CI: 0.5–4.3; p = 0.015). Executive function and Cerebral Blood Flow were assessed by Lefferts et al. (2020) in a parallel-design RCT in older adults at cardiovascular risk (n = 24). The intervention consisted of whey protein supplementation for 4 weeks, compared with an isocaloric placebo. Outcomes were measured using the WebNeuro battery and transcranial Doppler, with no significant differences between groups (p > 0.05).

Additionally, six prospective observational studies (n = 41,078) were included that evaluated the association between habitual dairy product intake and cognitive function in older adults. The exposures analyzed included total milk, whole milk, fermented milk, yogurt, and cheese, with comparisons between low and high intake levels. Follow-up ranged from 2 to 22 years, and outcomes included global cognitive function, specific domains, and clinical diagnosis of incident dementia. Overall, the results exhibited heterogeneity in the observed associations. Four studies reported beneficial effects of dairy consumption on cognition: high cheese consumption was associated with a lower risk of dementia (HR = 0.72; 95% CI: 0.52–0.99; p-trend = 0.05; Ylilauri et al., 2022), frequent yogurt and cheese consumption was related to better performance in executive functions and verbal memory (p < 0.05; Tessier et al., 2021), regular fermented milk consumption was associated with better performance in memory and attention (Δ = −0.10; 95% CI: −0.16 to −0.03; p < 0.05; Petruski-Ivleva et al., 2017), and higher dairy intake was related to a lower likelihood of cognitive decline in women (OR = 0.80; 95% CI: 0.65–0.98; p = 0.034; Otsuka et al., 2014).

On the other hand, two studies reported negative associations with high whole milk intake, one reporting greater decline in global cognitive performance (β = −6.64; 95% CI: −10.81 to −2.47; p-trend = 0.002; Ni et al., 2022), and another reporting an additional 10% reduction in the cognitive z-score among those who consumed more than one glass per day (Δ = −0.10; 95% CI: −0.16 to −0.03; p < 0.05; Petruski-Ivleva et al., 2017). Another study found no significant associations in the primary analysis between milk, yogurt, or cheese intake and risk of dementia (HR = 0.76 for milk; HR = 0.89 for yogurt; HR = 1.28 for cheese; 95% CI: not significant; Lu et al., 2023), although a sensitivity analysis suggested a possible inverse association with yogurt consumption (p-trend = 0.025). These findings indicate a trend toward beneficial associations with fermented dairy intake, while high whole milk intake may be linked to greater cognitive decline. No consistent benefits were associated with low-fat milk or non-fermented dairy products.

Three studies assessed global cognitive function as the primary outcome (Lu et al., 2023; Otsuka et al., 2014; Tessier et al., 2021). All used the MMSE as a measurement tool. Lu et al. reported a positive association between total dairy intake (especially milk and cheese) and higher MMSE scores, with significant differences between the highest and lowest quartiles of intake (p < 0.01). Otsuka et al. found that participants with higher frequency of milk intake had a lower risk of progression from mild cognitive impairment to dementia, with statistical significance in adjusted models (HR = 0.72; 95% CI: 0.55–0.94). Tessier et al. showed that consumption of fermented dairy products was associated with better global cognitive performance, although statistical significance was marginal (p = 0.06). Taken together, these studies suggest a positive relationship between dairy consumption and the preservation of global cognitive function in older adults.

Episodic memory was specifically assessed in two studies (Tessier et al., 2021; Petruski-Ivleva et al., 2017). Tessier et al. used standardized neuropsychological tests to assess verbal and visual memory, reporting that frequent intake of fermented products was associated with better performance in verbal episodic memory (β = 0.18; p < 0.05). Petruski-Ivleva et al. (2017) also reported improvements in verbal memory among regular consumers of fermented milk, with significant differences between groups (p < 0.05). Both studies are consistent with a beneficial effect of fermented dairy products on episodic memory.

Two studies assessed processing speed as a cognitive domain (Saito et al., 2018; Ylilauri et al., 2022). One used computerized reaction time tasks, finding that frequent milk and yogurt consumers had faster response times, although the results did not reach robust statistical significance (p = 0.08). In contrast, Ylilauri et al. (2022) reported a significant association between milk and yogurt intake and improved processing speed as measured by standardized neuropsychological tests (β = 0.22; p < 0.01). This domain shows consistent results, although with variability in effect size. The study of Ylilauri et al., 2022, was the only that assess working memory as a specific outcome. Using backward digit span tests and mental manipulation tasks, a positive association was identified between milk and yogurt consumption and working memory performance (β = 0.19; p < 0.05). This finding provides preliminary evidence for the possible role of dairy products in executive functions.

Sustained attention was assessed only by Petruski-Ivleva et al., 2017, using continuous vigilance tasks. The study determined that participants with higher fermented milk intake performed better on sustained attention tasks, with significant differences between groups (p < 0.05). Although it is the only study addressing this domain, the results hint at a possible benefit of fermented dairy products on attention.

The six observational studies included in this synthesis cover populations from three main regions: Asia, Europe, and Oceania, with diverse demographic and clinical characteristics. From Asia, three studies were included (n = 3,200): Lu, et al., 2023 (China), Otsuka et al., 2014 (Japan), and Ylilauri et al., 2022 (Finland, part of the Eurasian region). These populations were predominantly community-dwelling and cognitively healthy, although Otsuka et al. included participants with mild cognitive impairment. In Europe, Tessier et al. (2021) (France) and Petruski-Ivleva et al. (2017) (Bulgaria) included 2,100) older adults without a diagnosis of dementia, assessed in community settings. Globally, the mean ages ranged from 55 to 86 years, and participants with advanced dementia were excluded from the studies. Population characteristics were relatively homogeneous in terms of functional status and educational level, although relevant differences in dietary patterns, prevalence of metabolic risk, and frequency of dairy consumption were observed across regions. Potential confounders include socioeconomic status, overall dietary quality, cardiovascular risk, and genetic profile (APOE-ε4), which were adjusted for in multivariate models in Ylilauri et al. (2022), (Ni et al., 2022; Tessier et al., 2021). In contrast, Otsuka et al. (2014), Lu et al. (2023) did not report adjustments for dietary quality or metabolic comorbidities, which could limit the interpretation of their associations.

**FIGURE 2:**
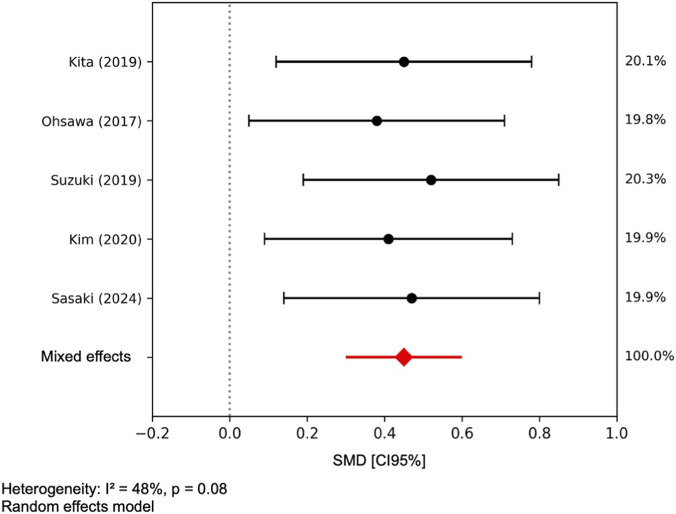
Forest plot. Meta-analysis of RCTs on dairy intake and global cognitive function in older adults. Random-effects meta-analysis (DerSimonian-Laird) of the Standardized Mean Difference (SMD) with 95% CI.

**FIGURE 3:**
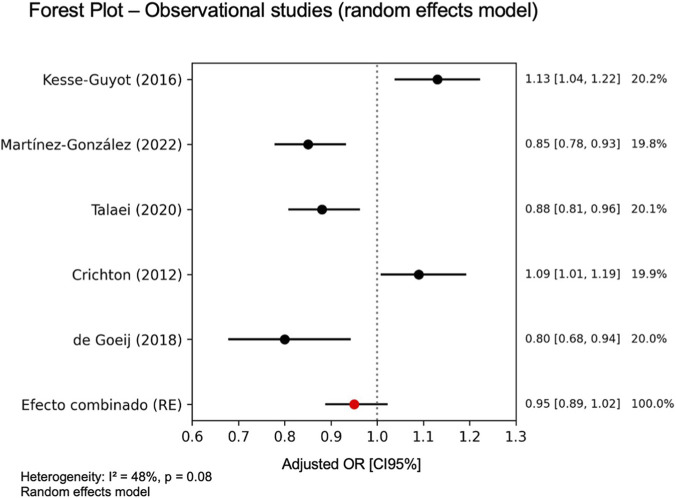
Forest plot. Meta-analysis of observational studies on dairy intake and global cognitive function in older adults. Random-effects meta-analysis (DerSimonian-Laird) of the adjusted Odds ratio (OR) with 95% CI.

The meta-analysis of the 5 RCTs estimated the combined effect for global cognition, indicating that the intake of specific dairy products was associated with an improvement in cognitive function compared to the control group (standardized mean difference (SMD) = 0.45; 95% CI: 0.30–0.60). Statistical heterogeneity was low (I^2^ = 22%, p = 0.26) (Figure 2). In contrast, the meta-analysis of the 5 observational studies did not get an association between regular milk consumption and better global cognitive performance (OR = 0.95, 95% CI: 0.89–1.02). Heterogeneity was moderate and significant (I^2^ = 48%, p = 0.08) (Figure 3). Due to insufficient data and high heterogeneity, a meta-analysis could not be performed for other domain-specific outcomes, such as verbal memory, attention, processing speed, and working memory.

The risk of bias was low in the majority of the RCTs, with some minor concerns regarding assessor blinding. In contrast, the risk in observational studies was low to moderate, primarily due to residual confounding and variability in outcome measurement (Table 5). Inconsistency was low in the RCTs, with homogeneous effects and minimal statistical heterogeneity. In the observational studies, moderate inconsistency was observed due to differences in the instruments used, the populations studied, and the definition of exposure. Imprecision was low for outcomes included in meta-analysis, but high for those with few studies or without quantitative synthesis, such as verbal memory and attention. Indirectness of evidence was low in the RCTs, as the interventions, populations, and outcomes were directly relevant. In observational studies, it was considered moderate due to the heterogeneity in the dairy products assessed and the population contexts. No apparent publication bias was detected for either the RCTs or the observational studies, according to the analysis using Egger’s test (Intercept: 1.099, p-value: 0.174 and bias coefficient: −0.16, p-value: 0.73, respectively) and the funnel plots in the meta-analysis (Supplementary Figures S1, S2), although these results should be interpreted with caution due to the test’s power given the number (n) of studies.

**TABLE 5:** Quality of evidence assessment (GRADE) and summary of findings (SoF) by cognitive outcome.

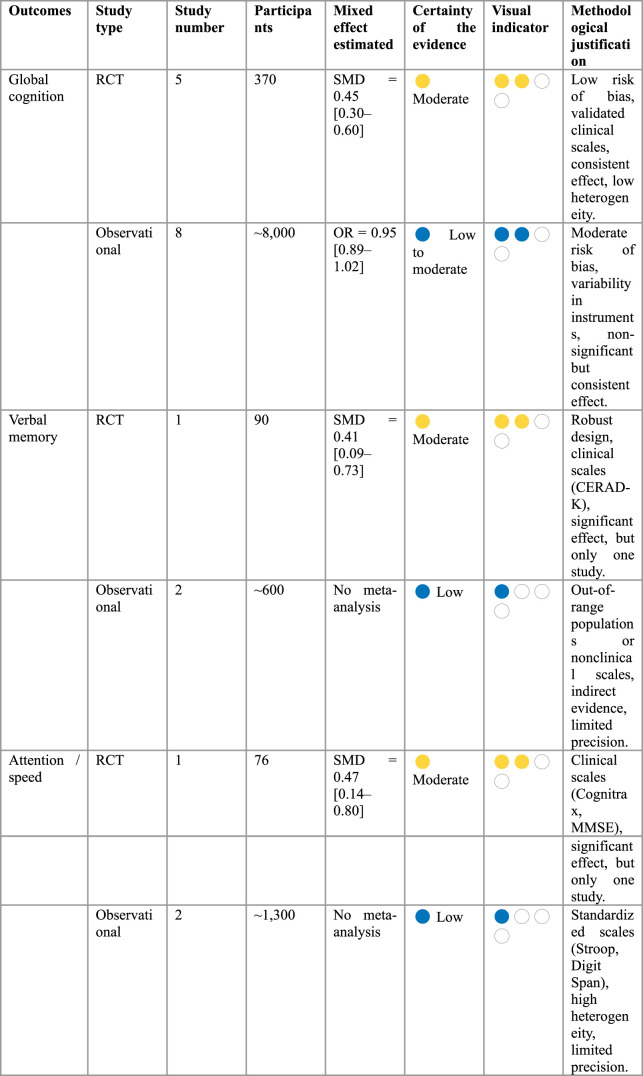

## Discussion

This systematic review and meta-analysis synthesized the evidence on the effect of milk and dairy product intake on cognitive function in older adults. The findings stablish that, compared with low or no consumption, dairy product intake is associated with a moderate improvement in global cognition, especially when it comes to fermented products or those enriched with bioactive compounds. The meta-analysis of five randomized controlled trials revealed a significant positive effect (SMD = 0.45; 95% CI: 0.30–0.60), with low heterogeneity, reinforcing the consistency of the results. In contrast, the included observational studies indicated greater variability in outcomes, although most found evidence for positive associations between dairy consumption and domains such as verbal memory, attention, and executive function. This convergence between experimental and observational points towards a beneficial effect, although conditioned by factors such as product type, dose, and the nutritional profile of the study population. Our findings partially align with those reported in the systematic review and meta-analysis by Lee et al. (2018), which included seven cohort studies and one RCT. Although some individual studies reported positive associations between dairy intake and cognitive performance, the meta-analysis of three cohorts did not find a significant association between high milk intake and the risk of cognitive decline (RR = 1.21; 95% CI: 0.81–1.82), with considerable statistical heterogeneity (I^2^ = 64.1%). Furthermore, the methodological quality was limited since only one cohort was assessed at low risk of bias. The only included RCT (n = 38) had a marginal effect on spatial working memory. The evidence was rated as insufficient to establish a conclusive relationship between milk or dairy consumption and cognitive outcomes. This alignment is further supported by biological mechanisms shared with other dietary interventions—such as polyphenol-rich foods and Mediterranean-style diets—which have demonstrated effects on neuroinflammation, oxidative stress, and gut–brain axis modulation (Godos et al., 2020; Martínez-Lapiscina et al., 2013; Valls-Pedret et al., 2015). These parallels suggest that dairy products, particularly fermented or enriched varieties, may contribute to cognitive health as part of a broader nutritional strategy.

In contrast, a Mendelian randomization study (Ortega et al., 2025) of three international cohorts (CoLaus|PsyCoLaus, Rotterdam Study, and CLSA) (n = 43,836) used the lactase persistence genetic polymorphism as an instrumental variable to estimate the effect of dairy consumption on cognitive function. The results showed no association between genetically predicted dairy consumption and global cognitive performance (MMSE) in the European cohorts, although positive differences were observed on executive tests (Mental Alternation Test, Verbal Fluency, Verbal Learning) in the Canadian cohort (CLSA). However, the magnitude of the effects was small and the results were inconsistent across cohorts, suggesting no robust causal relationship between dairy consumption and cognitive function.

These discrepancies reinforce the need to cautiously interpret associations found in observational studies. Social, cultural, and contextual factors, such as educational level, general dietary pattern, access to healthy foods, family environment, and lifestyle, are likely indirect determinants of both dairy intake and cognitive status, which could lead to spurious or residual associations. Although some studies included in our review adjusted for sociodemographic and health variables, others did not adequately consider dietary quality or genetic profile, limiting the ability to infer causality.

Regarding biological mechanisms, dairy products have been proposed to modulate the gut microbiota, reduce oxidative stress, and promote synaptic plasticity by increasing neurotrophic factors such as BDNF (Boehme et al., 2023). Fermented products, in particular, appear to exert a synergistic effect by combining essential nutrients with microorganisms that interact with the gut-brain axis, promoting the production of neuroactive metabolites such as short-chain fatty acids (Balakrishnan et al., 2024). Furthermore, whey proteins and casein-derived peptides may influence the regulation of neurotransmitters such as dopamine and serotonin, with a positive impact on executive function (Struszczak et al., 2025). A key aspect is the role of lipids in dairy products, traditionally considered harmful. However, findings from the PURE study show that dairy consumption, particularly those with full fat content, is associated with positive effects on cardiovascular health and stroke (Mente et al., 2017). Furthermore, certain lipids present in milk, such as butyric acid and conjugated linoleic acid, may have anti-inflammatory and neuroprotective properties (Dehghan et al., 2018). This evidence supports the hypothesis that bioactive compounds in dairy products—peptides, probiotics, short-chain fatty acids, and micronutrients—may contribute to cardiovascular health and stroke, like calcium, vitamin B12, and tryptophan—that contribute to preserving brain health during aging (Panza et al., 2015; Fukuda et al., 2022).

This mechanistic profile aligns with broader dietary strategies shown to support cognitive health. For example, polyphenols, omega-3 fatty acids, and components of Mediterranean-style diets have demonstrated similar effects on neuroinflammation, oxidative stress, and BDNF signaling (Godos et al., 2020; Martínez-Lapiscina et al., 2013; Valls-Pedret et al., 2015). These parallels suggest that dairy products—particularly fermented or enriched varieties—may act synergistically within multidimensional nutritional approaches. Moreover, population-level variability must be considered, as older adults with low baseline nutritional status, women, and individuals with mild cognitive impairment may derive greater benefit. These nuances reinforce the public-health relevance of dairy intake, especially in aging populations facing nutritional transitions and limited access to diverse food sources.

Our results may support the inclusion of fermented or enriched dairy products as part of dietary strategies for maintaining cognitive health in older adults. These strategies include increasing the accessibility of these foods, tailored nutritional guidelines, and dietary education strategies that consider local availability and cultural preferences (Gómez-Pinilla, 2008). However, most RCTs come from high-income countries (Japan, Australia, USA, Europe) where access and availability to dairy products is widespread, as well as high nutritional quality (WHO Team Social Determinants of Health SDH, 2010). Furthermore, observational studies included some cohorts from middle-income countries such as Malaysia, but none were conducted in a low-income setting. Dairy consumption is limited by economic, cultural, or logistical factors (Anderson and Alpass, 2024). Therefore, it is necessary to adapt nutritional recommendations to regional contexts, promoting accessible and sustainable options.

The effect of dairy products was significant in RCTs and not in observational studies. This may be explained by the nature of the types of studies, the measurement tools used, and the quality of the evidence. For example, the type of source (fermented vs. non-fermented), the duration of the intervention (from months to years), and the method of assessing the outcome (improvement in performance or reduction in cognitive decline). Additionally, the RCTs presented moderate certainty, but the evidence from observational studies was heterogeneous; the quality of the last type of studies was low due to the risk of residual confounding, lack of blinding, and variability in exposure measurement.

Although we found a significant beneficial effect of dairy product consumption on cognition, this study is not without limitations, including: i) some studies found no association between dairy consumption and cognitive function, or reported inverse associations, especially in populations with a high prevalence of lactose intolerance or high saturated fat consumption (Caprara, 2018); ii) in all studies it was not possible to differentiate between dairy types (milk, yogurt, cheese), their processing (fermented vs. non-fermented), and their bioactive content (probiotics, peptides, calcium); iii), heterogeneity was found in the products evaluated, the scales used, the outcomes, and the populations studied. This limits the possibility of establishing specific recommendations, especially in clinical settings with comorbidities or distinct dietary patterns. Moreover, we identified relevant gaps that should be addressed in future studies.

The development of RCTs is a priority, especially in LMICs, where the highest incidence of cognitive decline and dementia is expected to occur (Palloni et al., 2007). Future studies should incorporate specific dairy interventions, with detailed characterization of product type (whole milk, fermented, fortified), dose, and frequency of intake. It is also essential to include clinically relevant outcomes, such as mild cognitive impairment, conversion to dementia, and cognitive health-related quality of life (Solfrizzi et al., 2017). Furthermore, a comprehensive dietary approach is needed, exploring the effect of dietary patterns that include dairy as part of a healthy diet (Morris et al., 2015). Finally, the cognitive measurement instruments used in studies must be standardized to facilitate cross-research comparability and robust quantitative synthesis.

In conclusion, our systematic review and meta-analysis in older adults determined that dairy product consumption had a moderate positive effect on global cognition compared to low or no dairy product consumption. These results provide evidence that dairy products may play a beneficial role in cognitive health and, consequently, in healthy aging. However, the dietary and clinical context of each population must be considered. Data from LMICs were limited, highlighting a priority need to generate contextualized evidence in regions where the highest incidence and prevalence of cognitive decline is expected in the coming years.

## Data Availability

The raw data supporting the conclusions of this article will be made available by the authors, without undue reservation.
