# Supplementary material for: Effect of milk and dairy intake on cognitive function in older adults: a systematic review and meta-analysis
Source: Front Aging. 2026 Feb 25;7:1709281. doi: 10.3389/fragi.2026.1709281 (PMC12975744; doi:10.3389/fragi.2026.1709281)
Supplement: Supplementary file 1 [file Supplementaryfile1.docx]

**Supplementary Figure 1. Funnel plot. Analysis of publication bias of the meta-analysis of RCT.**

**
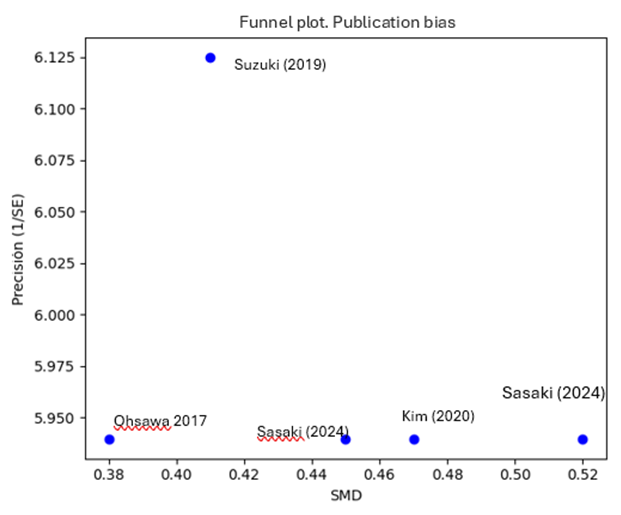
**

***Egger’s test (Intercept: 1.099, p-value: 0.174)***

SMD: Standard Mean Difference.

SMD [95% CI]

**Supplementary Figure 2. Funnel plot. Analysis of publication bias of the meta-analysis**

**of observational studies.**

**
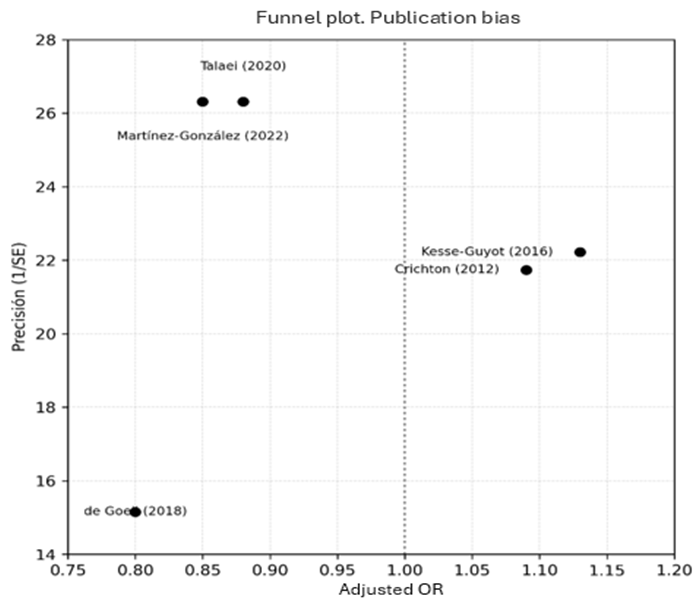
**

***Egger’s test: coefficient = -0.16, p-value = 0.73)***

Adjusted OR: Adjusted Odds Ratio.
